# ColorEM: analytical electron microscopy for element-guided identification and imaging of the building blocks of life

**DOI:** 10.1007/s00418-018-1707-4

**Published:** 2018-08-17

**Authors:** Nicole M. Pirozzi, Jacob P. Hoogenboom, Ben N. G. Giepmans

**Affiliations:** 1 Department of Cell Biology, University Medical Center Groningen, University of Groningen, Groningen, The Netherlands; 2 Department of Imaging Physics, Delft University of Technology, Delft, The Netherlands

**Keywords:** EDX, EDS, EELS, CL, NanoSIMS, ColorEM

## Abstract

Nanometer-scale identification of multiple targets is crucial to understand how biomolecules regulate life. Markers, or probes, of specific biomolecules help to visualize and to identify. Electron microscopy (EM), the highest resolution imaging modality, provides ultrastructural information where several subcellular structures can be readily identified. For precise tagging of (macro)molecules, electron-dense probes, distinguishable in gray-scale EM, are being used. However, practically these genetically-encoded or immune-targeted probes are limited to three targets. In correlated microscopy, fluorescent signals are overlaid on the EM image, but typically without the nanometer-scale resolution and limited to visualization of few targets. Recently, analytical methods have become more sensitive, which has led to a renewed interest to explore these for imaging of elements and molecules in cells and tissues in EM. Here, we present the current state of nanoscale imaging of cells and tissues using energy dispersive X-ray analysis (EDX), electron energy loss spectroscopy (EELS), cathodoluminescence (CL), and touch upon secondary ion mass spectroscopy at the nanoscale (NanoSIMS). ColorEM is the term encompassing these analytical techniques the results of which are then displayed as false-color at the EM scale. We highlight how ColorEM will become a strong analytical nano-imaging tool in life science microscopy.

## Introduction

Life is regulated by molecules that are organized in functional units, such as macromolecular complexes, structures, organelles, cells, and tissues. Microscopy is a highly important technique to study these building blocks and help to understand the underlying mechanism of normal life and disease. Electron microscopy (EM) can visualize the full structural complexity of tissue at nanometer (nm) resolution. However, the images are monochromatic and require labor-intensive analysis. Expert analysis is still subjective and localization of specific biomolecules is restricted to labeling, with typically up to three targets detected (de Boer et al. 2015), although five targets have been discriminated in a tour-de-force (Philimonenko et al. 2014). The recent Nobel Prizes awarded to advancements in microscopy underscore the importance of visualizing biomolecules at high resolution: GFP for live cells (2008; Martin 2009), breaking the diffraction limit with super-resolution fluorescence light microscopy (2014; Hell 2015) and single-particle cryo-EM (2017; Frank 2018).

While these groundbreaking techniques all contribute to a better understanding of biomolecule function, the identification of biomolecules in situ at a scale comparable to their size, i.e. in the multi-nanometer range, remains a crucial challenge. Correlated light microscopy and electron microscopy [CLEM; reviewed in de Boer et al. (2015)] allows to identify molecules and organelles based on fluorescence while maintaining contextual ultrastructure via EM. However, interpretation of the correlative images is hampered by the two orders of magnitude resolution gap that arises from the photons used (~ 400–700 nm).

**Fig. 1:**
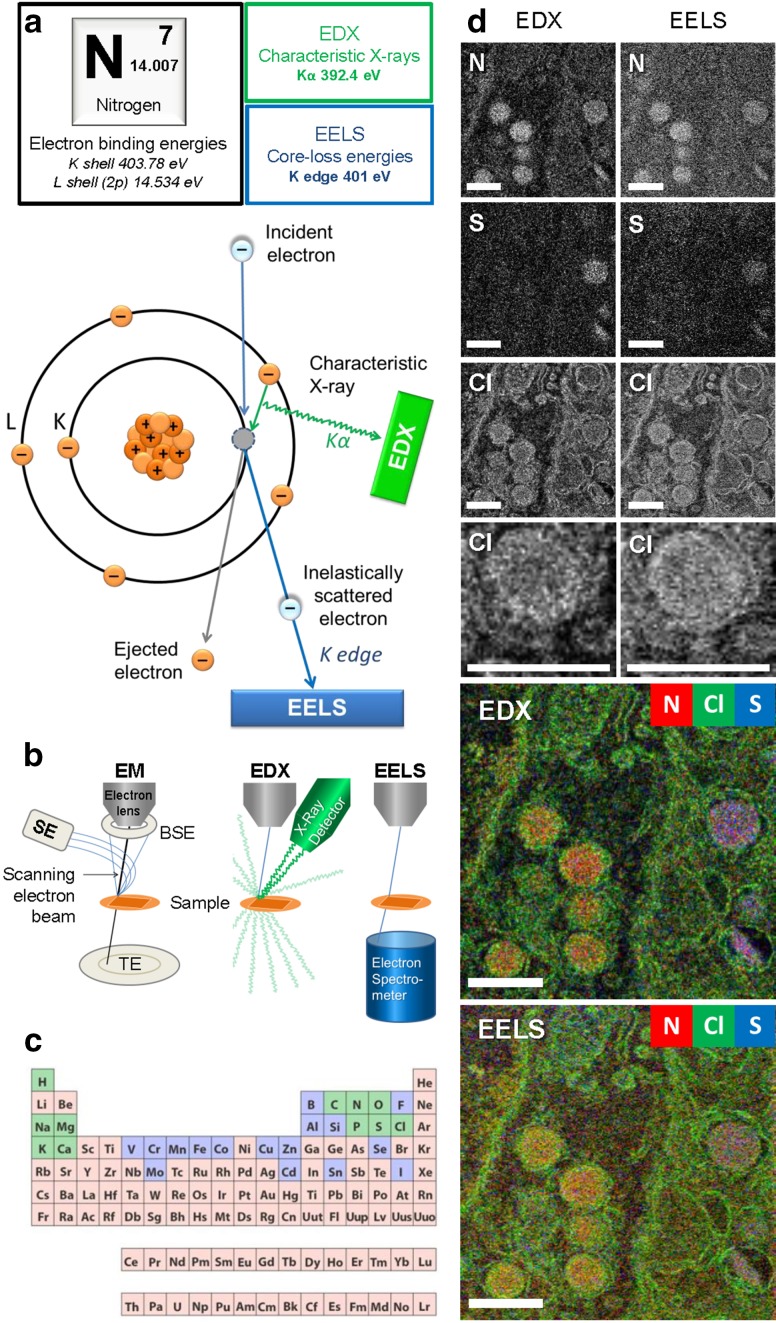
EDX and EELS physical principles and ColorEM. **a** The atomic origin of EDX and EELS signals is indicated for a nitrogen (N) atom. N has seven electrons arranged in two orbital shells: K and L. Electron binding energies of specified orbitals are shown (top). When an incident electron dislodges an electron from the K shell, inner-shell ionization occurs (bottom). To fill the unstable vacancy, an electron from the L shell drops and the difference in binding energies is released as a characteristic X-ray. A K shell vacancy being filled from an L shell electron results in a Kα line. This Kα X-ray is used to fingerprint N in the collected spectra. The incident electron that created the vacancy has lost an amount of energy approximately equal to the binding energy of the ejected electron. Therefore, in the EELS spectrum, transmitted electrons that have lost 401 eV represent N content in the sample. The zero-loss peak represents transmitted electrons that did not lose energy, and may be used to determine the sample thickness. Ionization edges represent the inner-shell ionization of sample atoms. **b** Arbitrary microscope configurations of EM, EDX, and EELS. Note that with EELS, the spectrometer must be at the transmission side of the sample, for EDX this is typically not the case. **c** Key to analytical EM is that most elements of the periodic system can be detected and identified. Elements in green are commonly found in the human body, those in green are present in trace amounts and those in pink are not found. **d** EDX and EELS analysis of EPON-embedded rat pancreas, where nitrogen (N), sulfur (S), and chlorine (Cl) maps are shown. Field of view contains heterochromatin in the nucleus, alpha cell glucagon-containing secretory granules, a cell border and then insulin-containing granules of the adjacent beta cell. While comparable, EDX maps show a better signal-to-noise ratio and EELS maps have greater detail as seen in the Cl map of one alpha cell granule. EELS analysis retained more membrane detail than EDX, which is expected because X-rays can be produced from a larger interaction volume. While EELS analysis was restricted to the elements mentioned, EDX analysis also obtained maps for phosphorus, osmium, oxygen, and more. The microscope was optimized for EELS acquisition, showing the robustness of EDX, able to produce similar maps with sub-optimal settings. Chlorine is typically seen in the osmicated regions of EPON-embedded tissue which is an artefact of embedding as opposed to endogenous content. Simultaneous analyses performed with 256 by 256 pixels, 900 µs dwell time, 200 keV, 6 nA, with EELS recorded from 66 to 570 eV. **c** Reproduced from https://askabiologist.asu.edu/content/atoms-life. Bars 0.5 µm

The use of analytical signals that originate from electron beam irradiation to obtain high spatial resolution compositional information of materials visualized in microscopic images has been explored since the early days of EM. Quantitative spectroscopic elemental analysis techniques, well established in the materials sciences, such as electron energy loss spectroscopy (EELS) and energy dispersive X-ray spectroscopy (EDX), have been explored for decades in biology. Recent advancements in detector sensitivity, computational power, and integration of different systems pave the way for broader implementation in the life sciences. Other analytical techniques, like cathodoluminescence (CL) or secondary ion mass spectrometry (SIMS), allow imaging at higher resolution and higher sensitivity through the development of smaller probe sizes and more sensitive detectors. The analytical information, obtained in gray-scale, can be displayed in false-color and then overlayed or added to the traditional EM images, creating ‘ColorEM’ (Barfels et al. 1998; Fig. 1). Here, we introduce the main concepts and operating principles of EDX, EELS, CL, and NanoSIMS, the hardware involved, and pioneering work in the life sciences in the past decade. Emphasis will be on the guidance of elements present in biosamples, which may be used to identify abnormalities, or other elements used as probes to identify specific targets. We focus on the future potential for nanoscale analytical imaging using each of these techniques to better identify subcellular structures and biomolecules to help to understand healthy life and diseases.

## Electron energy loss spectroscopy (EELS)

EELS is an analytical tool that not only can reveal elemental composition, but also several physical and chemical properties of a sample (Egerton 2011). When an electron in the electron beam interacts with the sample, many different processes can give rise to a small loss of energy. The amount of energy lost demonstrates both the nature of the interaction and the material with which interaction took place. The transmitted electrons are collected and analyzed at high spectroscopic resolution to reveal these energy losses (Fig. 1). An incident electron that ionizes an atom loses energy approximate to the binding energy of the dislodged electron (Fig. 1a). EELS spectra includes ionization edges, or core-loss energies, as well as fine details that provide information regarding binding stacks, coordination, oxidation, and more. For each ionization edge, the loss of energy is unique for the atom or molecule that was ionized. Specific EELS signatures are indicative of the atomic composition of the sample but, because of the unique loss patterns for each element, analysis is laborious. Electron energy losses of 1.6–3.4 eV correspond to interactions that give rise to the emission of light in the visible spectrum (Mhawi 2009). EELS signatures in this energy range allow detection and localization of chromophores within the EM image (Barfels et al. 1998). The ionization interaction in which incident electrons lose energy, determined by EELS, will also give rise to the emission of X-rays that can be detected with EDX (Fig. 1a). Collected electrons are all transmitted through the sample (Fig. 1b), requiring the electron spectrometer to be on the transmission side of the sample and giving this elemental analysis high spatial  resolution and sensitivity. Samples for EELS should be ultrathin since only one inelastic scattering event at most, should occur per transmitted electron. For more detailed information regarding the fundamentals of EELS, see Hofer et al. (2016) and the extensive book by Egerton (2011).

EELS data can be obtained using two distinct ways: energy-filtered transmission EM (EFTEM) and scanning transmission EM EELS (STEM-EELS; Aronova and Leapman 2012). With EFTEM, all transmitted electrons are filtered based on their amount of energy loss, forming an image with only electrons having undergone the same interaction, indicative of a specific element or molecule in the sample. EFTEM is compatible with standard TEM operation and can capture large fields of view at once. However, with  only one small energy loss window, only one element can be measured at a time. With STEM-EELS, a focused electron beam scans the sample and all transmitted electrons are collected and spectroscopically analyzed. In this manner, a larger energy loss spectrum is recorded on a pixel by pixel basis and afterwards certain energy losses can be selected denoting certain elements or molecules (Aronova and Leapman 2012). EELS data shown in Fig. 1d were acquired with STEM-EELS in a spectrum of 66–570 eV, chosen to detect the elements shown. Direct electron detectors replacing charge-coupled devices (Maigne and Wolf 2018), recently provided a threefold improvement in the signal to noise ratio in EELS (Ramachandra et al. 2014).

Recent bioimaging of EELS includes water and sugar in frozen hydrated samples and the detection of different cargo-containing vesicles in pancreas (Aronova and Leapman 2012; Leapman 2017). EELS can show presence of nucleic acid components and proteins via phosphorus and nitrogen, in parallel with visualization of targeted quantum dot nanoparticles (Nisman et al. 2004) and the opportunity to detect lanthanides probing different targets (Adams et al. 2016). Thus, since the past decade, EELS analysis adds another dimension to specifically visualize probes and endogenous content based on elemental composition at (S)TEM resolution (Fig. 1).

## Cathodoluminescence (CL)

**Fig. 2:**
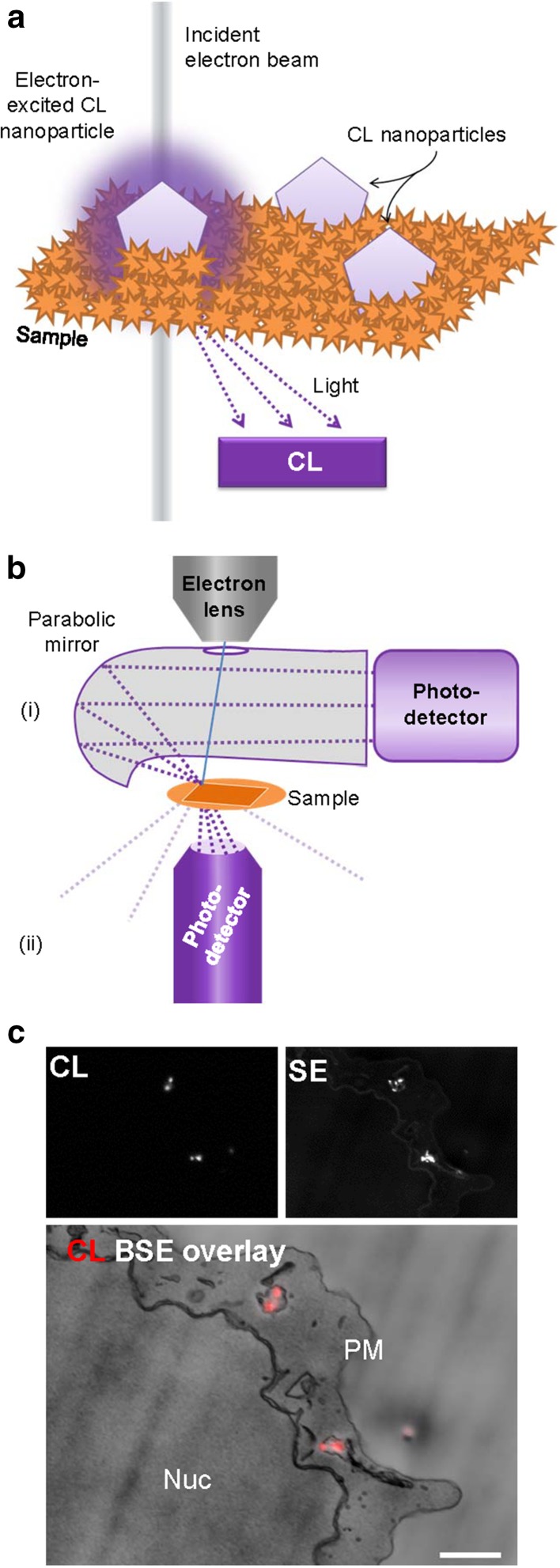
Cathodoluminescence principle and bioapplication. **a** Upon electron beam irradiation, some molecules and nanoparticles will emit light within the visible spectrum. The CL mechanism is an interaction with luminescent molecules or particles. With the raster scan of the electron beam, luminescence is recorded at each beam position, providing localization of the photon-emission in the EM image. **b** Electron microscope configurations of CL. The photodetector can either be combined with a (i) parabolic mirror, or (ii) a light objective and detector. **c** CL and SE images of internalized 40 nm nanodiamonds by cultured cells (EPON-embedded). The overlay of the CL and BSE images show the nanodiamonds in vesicles within the cell between the nucleus (Nuc) and plasma membrane (PM). Note that some nanodiamonds are visible in SE but did not show CL because of the presence of negative nitrogen vacancies and lack of neutral nitrogen vacancies. *SE* secondary electron detector, *BSE* backscattered electron detector. **c** Reproduced from Hemelaar et al. (2017). Bar 1 µm

CL is the emission of visible light after interaction with an electron beam. By collecting the emitted light, CL molecules or luminescent particles can be visualized in EM (Fig. 2). Light detection is mostly done with a parabolic mirror and photodetector but more recently also integrated light-electron microscopes are being used for CL (Fig. 2; Hemelaar et al. 2017; Nagayama et al. 2016; Narváez et al. 2013).

A fundamental challenge for CL, known from when it was first described in plants (Pease and Hayes 1966), is that the probability of generating visible light is very low. To increase signal, long exposure times can be used, but that leads to damage of the organic molecules and thus bleaches the natural luminescence prior to adequate image formation (Echlin 1971; Niitsuma et al. 2005); see Coenen and Haegel (2017) for extensive CL fundamentals and detection. As a consequence, the use of organic molecules, like the interesting CL detection from enhanced green fluorescent protein (Nagayama et al. 2016), might not lead to nanometer-range resolution imaging. In the past decade, the use of more electron-resistant nanoparticles as CL probes has been explored. Particles of yttrium oxide (Fukushima et al. 2014, 2016) and lanthanum trifluoride (Keevend et al. 2017) doped with lanthanides have CL emissions that can be tuned by controlling the type of lanthanide impurity. Nanodiamonds have been shown to have CL capabilities with different defect centers in the diamond emitting different color light (Nagayama et al. 2016). The detected particle-specific wavelength emissions can then discriminate targets, four distinct CL spectra simultaneously have been demonstrated (Glenn et al. 2012; Morrison et al. 2015; Niioka et al. 2014; Tizei and Kociak 2012). CL lifetime can be detected as an alternative or additional means to discriminate nanoparticle emissions (Garming et al. 2017). However, the current limitation of CL towards nanoscale resolution lies in the size of the nanoparticles, most of which are still way beyond the scale of target proteins (Fukushima et al. 2014; Hemelaar et al. 2017). If CL-proteins are developed from fluorescent proteins, retention of CL-properties after EM sample preparation and in the EM will be a challenge, similar to fluorescent proteins used for super-resolution microscopy (Paez-Segala et al. 2015) Thus, while CL has the potential benefit of discriminating multiple targets at high resolution and using different emission wavelengths, serious hurdles have to be taken to make CL-imaging a routine microscopic technique in life sciences. While the use of CL may be promising, application will be limited to a few targets and will only reach higher potential if a toolbox of smaller probes is developed (Prigozhin et al. 2018) and/or if current electron-induced damage thresholds can be overcome.

## Electron dispersive X-ray analysis (EDX)

EDX is an elemental composition analysis technique that can be easily performed on any existing scanning (transmission) EM with the addition of an X-ray detector. EDX utilizes specific radiation produced when the incident beam creates an electron vacancy in the sample (Fig. 1a). At the atomic level, electron vacancies created from the incident electron beam are quickly filled by electrons of higher energy shells, releasing radiation of the difference in electron binding energies in the form of characteristic X-rays. These characteristic X-rays are energy-specific to the atom from which they were produced and thus reveal the elemental composition of a sample. Scattered electrons can still have enough energy to cause atomic ionizations farther from the incident beam location, therefore, the spatial resolution of EDX is less than that of EELS, which only analyzes transmitted electrons. This difference is spatial resolution is shown in the chlorine maps (Fig. 1d). While most of the approaches use electron irradiation, creation of the electron vacancies can be achieved through any exposure to high-energy particles or radiation. For instance, particle-induced X-ray emission or proton-induced X-ray emission (PIXE; Budka et al. 2005; Carmona et al. 2010) and synchrotron X-ray fluorescence microscopy [SXRF or XRM; (Carmona et al. 2010; Finney et al. 2006)] using incident high energy X-rays have been employed for elemental mapping in biology. However, these approaches typically require more advanced equipment precluding routine application combined with EM and will not be further considered in this review. For a comprehensive background on X-ray principles, hardware and analysis for non-physicists we highly recommend the book by Friel et al. (2017).

EDX, like EELS, has been widely applied in geology and material science since the 1940s. The transition to life sciences has been marked by developments in X-ray detector technology. The first applications came in the 1970s after the introduction of energy-dispersive semiconductor-based detectors (Echlin 1971; Roomans and Von Euler 1996). New advances with silicon drift X-ray detectors make it possible for EDX to bridge to life sciences as lighter elements can now be measured with greater signal to noise ratios, and achieved theoretical resolution, independent of temperature and count rates (Friel et al. 2017). The next generation of detectors are annular, surrounding the sample and increasing the solid angle to more than 1 steradian which increases count rate and shortens acquisition time (Teng et al. 2018).

ColorEM of endogenous elements, such as the phosphorus enriched in membranes and DNA, nitrogen in polypeptides, and sulfur in methionine-rich and cysteine-rich proteins can be qualitatively measured, mapped, and overlayed using EDX. The additional dimension of the electron image aids identification, making analysis more objective and less interpretation-based. Different particles can be used as EM immunolabels and then differentiated on the basis of their elemental content, as seen with gold nanoparticles and quantum dots with a cadmium selenide core. Quantification of biological samples presents new challenges where even the estimation of the background signal required new considerations, stimulating new modeling (Roomans and Kuypers 1980). Thus, in life sciences, EDX progressed from whole cell spectra to organelle-specific spectra (Somlyo et al. 1977b), to full mapping with individual spectra collected in nm-scale pixels (Scotuzzi et al. 2017).

## Secondary ion mass spectrometry at the nanoscale (NanoSIMS)

**Fig. 3:**
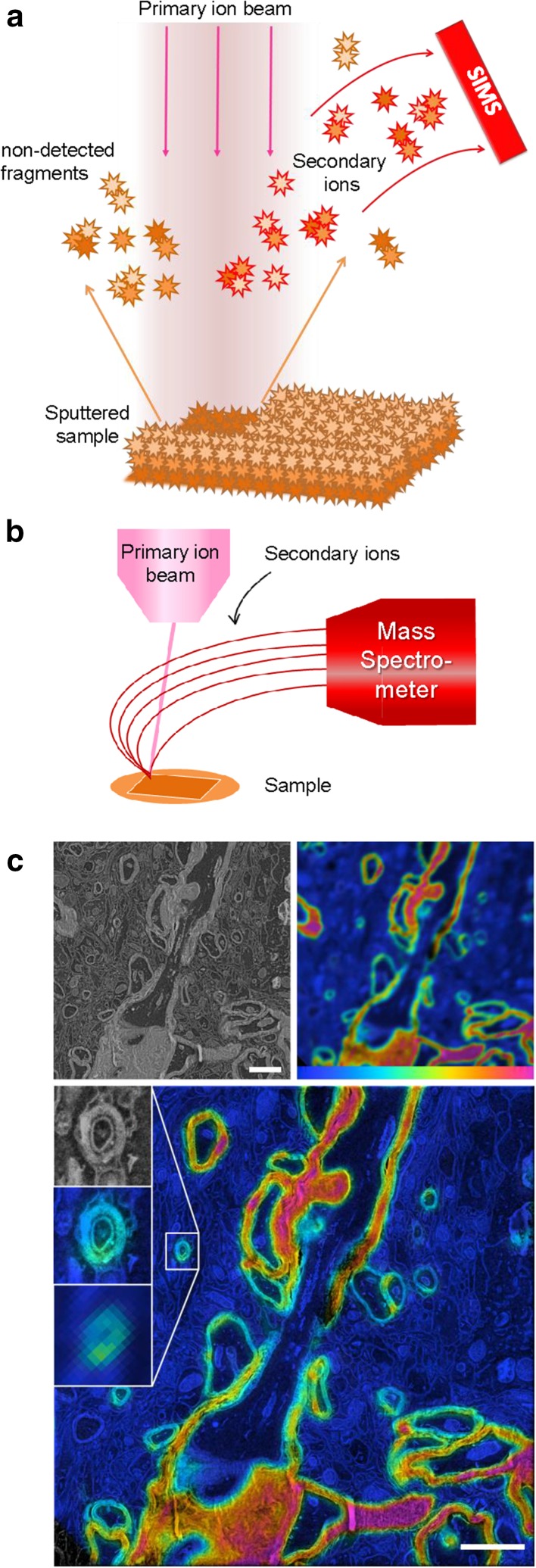
NanoSIMS in biology: From concept to application. **a** A highly focused primary ion beam scans the surface of the sample. Upon interaction sputtering occurs, dismantling surface atoms and molecules and ionizing some. These secondary ions are then drawn to a mass spectrometer by an electric field. The mass-based identified atoms/molecules can then be mapped to where the sputtering occurred. **b** Microscope configuration consisting of the primary ion beam scanning over the sample and secondary ions traveling to the mass spectrometer. **c** NanoSIMS used to image mouse brain labeled with isotopic nitrogen (^15^N) to study long-lived molecules and memory. The ratio between ^15^N and the naturally abundant ^14^N is shown in SIMS ranging from the natural occurrence (0.4% shown in blue) to enriched abundance (9% shown in red). The SEM and correlating SIMS image show a large disparity in resolution, acquired with a pixel size of 3.5 nm and 78 nm, respectively. With newly developed algorithms and image processing by Vollnhals and colleagues (2017), the SIMS data can be adjusted to correspond to the SEM data. Reproduced from Vollnhals et al. (2017). Bar 2 µm

Secondary ion mass spectrometry (SIMS) can sensitively measure atoms or atomic clusters ionized from spatially separate locations to achieve mass spectrometry imaging (Fig. 3; Svatoš 2010). A focused ion beam scans over a sample sputtering the material into neutral and ionized atomic and molecular components. The ionized components are collected and directed to a mass spectrometer, sensitively identifying the ions by their mass/charge ratio. Primary ion beams of oxygen or cesium are typically used with tendencies to generate positive or negative secondary ions, respectively. NanoSIMS is an emerging nanometer-scale imaging technique in biology (Jiang et al. 2016; Pett-Ridge and Weber 2012; Wirtz et al. 2015), with a lateral resolution of ~ 50–200 nm. Unlike CL, EDX, and EELS, NanoSIMS does not use the electron beam to generate content-specific signals, but instead uses an ion beam for the necessary etching. A secondary electron image can be detected from the ion beam, but not with the resolution achieved with an electron beam. To retrieve both SIMS and TEM information and generate composite images of structure and content, sequential sections can be imaged and merged (Lee et al. 2017), where thin sections for TEM (50 nm), and thicker sections for light microscopy/NanoSIMS (500 nm) can be used. Nearly all elements can be detected, but the relative sensitivity factor varies depending on the primary beam, and the element or molecule of interest (Wilson 1995). NanoSIMS can be used to examine isotope-labeled specimens which is generally known under the broader term nm-scale stable isotope probing [nanoSIP; Pett-Ridge and Weber (2012)]. NanoSIP allows tracking of protein turnover and cell renewal using molecules that are biologically identical to their non-isotopic counterparts (Jiang et al. 2016). Nuñez et al. (2018) reviewed the history, current use and expected improvements of NanoSIMS and its data analysis in more detail.

While NanoSIMS may not reach the spatial resolution achieved with electron beam imaging, the technique will allow imaging at the ~ 100 nm scale, has a high sensitivity and allows more complex discrimination of sample content than analyzing elements only. Another breakthrough may come from further development of mass spectroscopy imaging techniques that allow identification of specific biomolecules (Bodzon-Kulakowska and Suder 2016), however, also here the challenge is achieving spatial resolution at the nanometer scale.

## Which approach to choose from the new toolbox?

ColorEM allows visualization of biomolecules and ions directly, or by analysis of the elemental fingerprint of an organelle, vesicle, or other biological building block, where each technique has their characteristic benefits and limitations for bioapplications (Table 1). Moreover, exogenous molecules may highlight certain target proteins, like stable isotopes in NanoSIMS, and with nanoparticles in EELS, EDX, and CL. Also labels that are either genetically targeted or immunotargeted to molecules or organelles of interest can be highlighted with ColorEM. How one would proceed will depend on the research question and the type of sample, with the notion that ColorEM is an emerging technique that still may be challenging to implement. Guidance will be by taking pioneering work as a lead (Table 2). Examples and considerations for which technique to apply are detailed below.

**Table 1:**
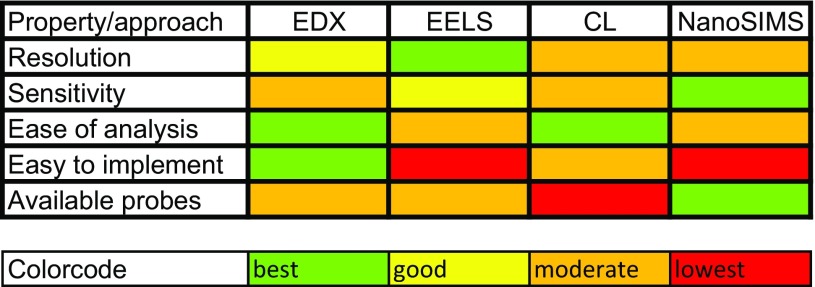
Feasibility of EDX, EELS, CL and NanoSIMS

A generic relative comparison of features of ColorEM summarized to consider prior to application in life science projects

**Table 2:**
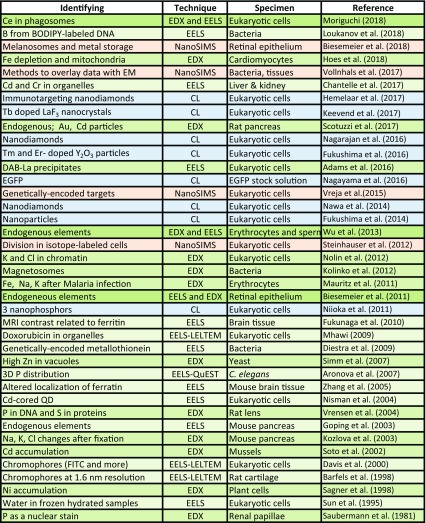
Guidance to choose the right analytical tools for ColorEM by examples of recent implementations of EELS, EDX, CL and nanoSIMS in biology

Pioneering studies proving the feasibility of analytical techniques in ColorEM. For an overview of EELS/EDX until 2007 the reader is referred to Fernandez-Segura and Warley (2008) and for a complete overview of nanoSIMS applications to Nuñez et al. (2018). See main text for details and abbreviations

### Endogenous detection

Biological material consists of mainly carbon but certain cellular features can be discriminated based on other elements present. DNA, protein, lipid and sugar all have a high carbon, hydrogen, and oxygen content, but may be discriminated by relatively high amounts of other elements. Concentrated proteins are hallmarked by their high nitrogen content, and in some cases sulfur for those proteins rich in cysteine and methionine, such as insulin visualized with EELS (Aronova and Leapman 2012; Goping et al. 2003) and EDX (Scotuzzi et al. 2017; Fig. 1). The condensed DNA in chromatin is rich in phosphorus and nitrogen (Saubermann et al. 1981; Vrensen et al. 2004). Early imaging of phosphorus and sulfur content, in conjunction of ions like iron, potassium, and zinc in mammalian cells to highlight the potential of EDX in bioimaging was demonstrated on a custom-designed microscope only 5 years ago, albeit with relatively larger pixel size (Wu et al. 2013). Similarly, the retinal pigment epithelium was analysed using ColorEM with both EELS and EDX and allowed discrimination between two subcellular bodies, namely melanosomes based on copper, and lipofuscin, based on phosphorus content (Biesemeier et al. 2011). Early CL experiments revealed signals from varied biomolecules, including cyclic amino acids (tyrosine, tryptophan) and nucleosides (Herbst and Hoder 1978) but CL analysis to identify DNA or amino acids has not become widespread. NanoSIMS analysis most advantageously focuses on isotope-labeled features (Jiang et al. 2016), but also is a good tool to identify ions, e.g. sodium (Na^+^), calcium (Ca^2+^) and iron (Fe^3+^; Biesemeier et al. 2018). When the biological question pertains to diffusible ions, cryo techniques must be employed (Zierold and Schäfer 1978) as explained below.

### Elements unique to certain cells

The interaction between the environment and organism assessed by the accumulation of environmental elements has been studied in a multitude of samples. Using unique signatures that are not naturally present results in a high signal to noise ratio when certain elements are locally accumulated (Table 2). In botany, EDX has been proven successful in analyzing accumulations of metals and metalloids [reviewed in van der Ent et al. (2018)], for example, nickel in plant cells (Sagner et al. 1998). In magnetotactic bacteria, membrane enclosed iron-containing crystals and correlating oxygen- or sulfur-rich inclusions have been imaged with EDX (Kolinko et al. 2012). In digestive cells of cadmium-exposed mussels, not only was cadmium detected, but notably, also silver and sulfur were enriched (Soto et al. 2002). The latter observation also shows the unbiased approach of EDX, with which analysis may lead to highly informative, unexpected results. In malaria infection, iron in red blood cells is decreased and the sodium/potassium ratio is increased as revealed using EDX (Mauritz et al. 2011). We now standardly apply EDX-based imaging when anomalies are found in electron images, this recently revealed that inclusion bodies found in iron-depleted cardiomyocytes are enriched in nitrogen and sulfur (Hoes et al. 2018).

### Stains and probes to specifically highlight molecules and organelles

Addition of ‘color’ to identify transport routes, organelles, and molecules in ColorEM is typically similar to other means of targeting in microscopy. In brief, particles can be taken up, cells can be stained, or targets can be identified using immunotargeting or genetic targeting.

### Nanoparticle uptake

Nanodiamonds with different coatings have been visualized with CL to determine different cellular uptake routes, namely clathrin-mediated endocytosis and micropinocytosis (Nagarajan et al. 2016). Several labs imaged CL successfully from internalized nanoparticles (Table 2), for instance nanoparticles doped with rare earth elements [erbium, thulium, ytterbium; Fukushima et al. (2016)] and terbium-doped lanthanumfluoride (LaF_3_) nanocrystals (Keevend et al. 2017).

### Immunotargeting

**Fig. 4:**
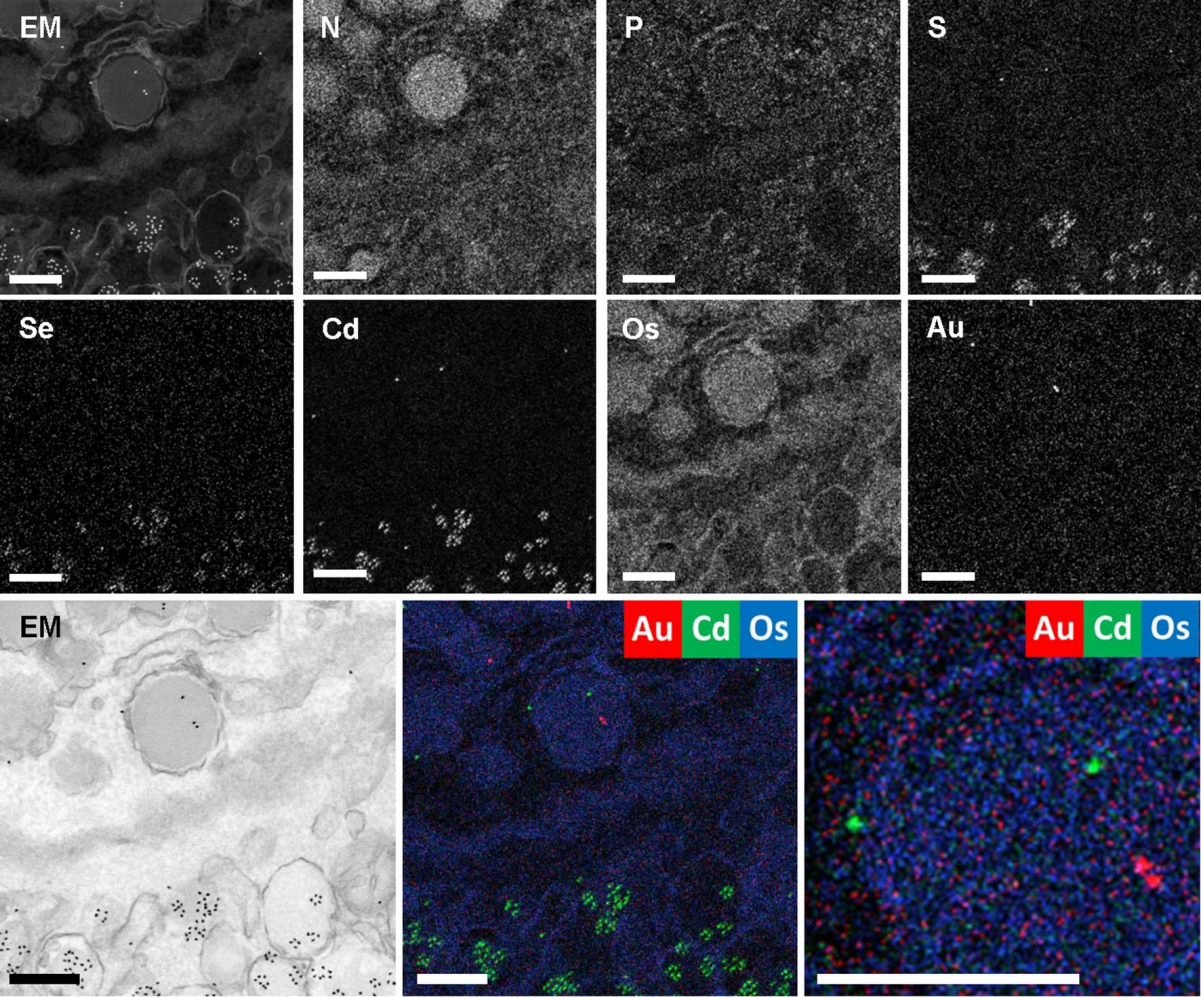
Nanometer-scale fingerprinting using endogenous signals, stains, and probes. EDX of EPON-embedded rat pancreas labeled for glucagon with 5-nm gold particles and insulin with CdSe-cored quantum dots 655. High angle annular dark field (HAADF; EM/inverted EM) image, elemental maps of nitrogen (N), phosphorus (P), sulfur (S), selenium (Se), cadmium (Cd), osmium (Os), gold (Au) and overlays as indicated. Note that EDX is a straightforward way to identify varied immunolabels where they are not easily discriminated in the HAADF image with this field of view. Bars 0.25 µm

Several groups showed proof-of-concept biofunctionalization of nanodiamonds (Glenn et al. 2012; Hemelaar et al. 2017a) and subsequent immunolabeling to be visualized with CL. However, these particles are typically relatively large (~ 40–200 nm), and their size excludes routine use to identify biomolecules at their scale (nm-range). Application of the smaller quantum dots, made up of, e.g. cadmium selenide, and gold particles may currently be the best option for successful targeting and analysis using EELS (Nisman et al. 2004) and EDX (Scotuzzi et al. 2017), as these are both routinely used in immuno-EM. The resulting analytical data no longer requires discrimination based on shape and electron-density, but the particles can be identified by elemental mapping, localizing multiple targets in the context of endogenous elements (Fig. 4).

### Genetic targeting

Genetically-encoded tags have the benefit over immunolabeling that, once the DNA is inside cells, it does not need destruction of the cells under study to target the labels. Recently lanthanides (lanthanum, cerium, praseodymium) have been complexed to diaminobenzidine (DAB) to allow elemental analysis of targets (Adams et al. 2016). The DAB polymers are typically made by probes that photo-oxidize or induce enzymatic reactions (Adams et al. 2016). Other candidates to generate accumulations of rare elements in biology include the ferritag as a probe that can be discriminated based on iron binding (Clarke and Royle 2016) and metallothionein used in combination with gold (Diestra et al. 2009). However, engineering and optimization of these emerging probes will be required before widespread implementation.

### Stains

Post-fixation with osmium tetroxide and contrasting with uranyl acetate and lead citrate are routine. These heavy metals are typically used for electron-dense contrast, but can now be separated based on elemental enrichment (osmium in Fig. 4). This also can be achieved with many stains explored for EM before, as long as these stains have a direct affinity for certain targets. These may include direct affinity probes containing elements not normally found in the human body (Fig. 1c) that visualize cancer drugs with EELS (Mhawi 2009) or stains that are deposited in a specific way depending on probes like the DAB-lanthanides discussed before (Adams et al. 2016). Similar success with cerium-DAB that is specifically phagocytosed and subcellularly analyzed with EDX and EELS has recently been demonstrated (Moriguchi 2018).

### Sample preparation

Fixation, either physical or chemical, is the first and most crucial step in preparing a specimen for EM and elemental analysis. The type of specimen is the first variable, here we focus on mammalian cells. Plant cells require other protocols in botany [reviewed by van der Ent et al. (2018)]. EELS and EDX spectroscopy and imaging on cryo-fixed tissue have been pioneered from the early days of analytical EM (Feng et al. 2004; Hall and Gupta 1982; Shuman et al. 1983; Somlyo et al. 1977a; Zierold and Schäfer 1978). The analysis of diffusible ions, is restricted to cryo-techniques because standard dehydration and embedding extracts much (if not all) of the elements of interest. Chemical fixation was found to increase phosphorus, sulfur, chlorine, and potassium and decrease calcium, iron, and zinc composition compared to cryo-fixed in murine brain tissue (Hackett et al. 2011), which obviously depends on the exact procedure and reagents being used. Cryo-fixed, cryo-sectioned, freeze-dried mouse cerebellum sections also have been used for EELS and EDX (Buchanan et al. 1993), and difference in extraction of ions depending on tissue has been reported (Hongpaisan and Roomans 1995). With the ability to analytically image EM samples routinely, a new evaluation of sample preparation, including the latest improvements on cryofixation techniques, will need to be performed to find the ‘best’ preservation protocol, which again will depend on the specific sample and question under investigation (Table 2). For X-ray collection applications, not only preservation of signal is required, but also preventing overwhelming signals from extraneous components, like copper grids. Composition of sample holder can make a large contribution to the specimen analysis. Since carbon is typically not discriminative in biosamples, carbon sample holders are commonly used in EDX analysis (Liljesvan and Roomans 1976).

## Outlook

20 years ago, Roomans and von Euler stated that “While geologists and material scientists started using the technique [EDX] in the late 1940s, biologists had to wait for serious work until around 1970, when a new, more efficient type of detector …” (Roomans and Von Euler 1996). We want to add that for routine imaging the 1970s was still too early, and only now with the current advancement in detector strategies, probe development, fixation approaches, and image data analysis a few labs now start to implement EDX and EELS as a further dimension to microscopy at the nanometer range resolution. EDX is the technique we feel is the most user-friendly regarding sample preparation, data acquisition, and analysis. With the restrictions of sample thickness and the need of precise spectral interpretation, EELS is only valuable when the higher resolution and sensitivity is required for the scientific question. CL mainly will be useful to look at particle uptake, and not to identify biomolecules, while NanoSIMS and other mass-based imaging technique are very sensitive, discriminating beyond single elements, but are not nearing the nanoscale yet. Further improvement of these techniques will lead to the ultimate goal: unbiased fingerprinting of subcellular content at the nanometer scale, the size range of biomolecules. However, this may not be reached within the next two decades because of the significant steps that need to be taken. The current ColorEM, however, already allows simultaneous identification of up to a dozen molecules, subcellular structures, and other organelles in a single image by analytical analysis, and since integration is straightforward in imaging centers, it will rapidly become common practice in the cell biologist community.

## References

[CR1] Adams SR, Mackey MR, Ramachandra R, Palida Lemieux SF, Steinbach P, Bushong EA, Butko MT, Giepmans BN, Ellisman MH, Tsien RY (2016). Multicolor electron microscopy for simultaneous visualization of multiple molecular species. Cell Chem Biol.

[CR2] Aronova M, Leapman R (2012). Development of electron energy-loss spectroscopy in the biological sciences. MRS Bull.

[CR3] Barfels MM, Jiang X, Heng YM, Arsenault AL, Ottensmeyer FP (1998). Low energy loss electron microscopy of chromophores. Micron.

[CR4] Biesemeier A, Schraermeyer U, Eibl O (2011). Chemical composition of melanosomes, lipofuscin and melanolipofuscin granules of human RPE tissues. Exp Eye Res.

[CR5] Biesemeier A, Eibl O, Eswara S, Audinot J, Wirtz T, Schraermeyer U (2018). Transition metals and trace elements in the retinal pigment epithelium and choroid: correlative ultrastructural and chemical analysis by analytical electron microscopy and nano-secondary ion mass spectrometry. Metallomics.

[CR6] Bodzon-Kulakowska A, Suder P (2016). Imaging mass spectrometry: instrumentation, applications, and combination with other visualization techniques. Mass Spectrom Rev.

[CR7] Buchanan RA, Leapman RD, O’Connell MF, Reese TS, Andrews SB (1993). Quantitative scanning transmission electron microscopy of ultrathin cryosections: subcellular organelles in rapidly frozen liver and cerebellar cortex. J Struct Biol.

[CR8] Budka D, Mesjasz-Przybyłowicz J, Tylko G, Przybyłowicz WJ (2005). Freeze-substitution methods for Ni localization and quantitative analysis in Berkheya coddii leaves by means of PIXE. Nucl Instrum Methods Phys Res Sect B Beam Interact Mater Atoms.

[CR9] Carmona A, Devès G, Roudeau S, Cloetens P, Bohic S, Ortega R (2010). Manganese accumulates within golgi apparatus in dopaminergic cells as revealed by synchrotron X-ray fluorescence nanoimaging. ACS Chem Neurosci.

[CR10] Clarke NI, Royle SJ (2016). FerriTag: a genetically-encoded inducible tag for correlative light-electron microscopy. bioRxiv.

[CR11] Coenen T, Haegel NM (2017). Cathodoluminescence for the 21st century: learning more from light. Appl Phys Rev.

[CR12] de Boer P, Hoogenboom JP, Giepmans BN (2015). Correlated light and electron microscopy: ultrastructure lights up!. Nat Methods.

[CR13] Diestra E, Fontana J, Guichard P, Marco S, Risco C (2009). Visualization of proteins in intact cells with a clonable tag for electron microscopy. J Struct Biol.

[CR14] Echlin P (1971). The application of scanning electron microscopy to biological research. Philos Trans R Soc Lond Ser B Biol Sci.

[CR15] Egerton RF (2011). Electron energy-loss spectroscopy in the electron microscope.

[CR16] Feng J, Somlyo AV, Somlyo AP (2004). A system for acquiring simultaneous electron energy-loss and X-ray spectrum-images. J Microsc.

[CR17] Fernandez-Segura E, Warley A (2008). Electron probe X-ray microanalysis for the study of cell physiology. Methods Cell Biol.

[CR18] Finney L, Mandava S, Ursos L, Zhang W, Rodi D, Vogt S, Legnini D, Maser J, Ikpatt F, Olopade OI, Glesne D (2006). X-ray fluorescence microscopy reveals large-scale relocalization and extracellular translocation of cellular copper during angiogenesis. Proc Natl Acad Sci USA.

[CR19] Frank J (2018). Single-particle reconstruction of biological molecules—story in a sample (nobel lecture). Angew Chem Int Ed Engl.

[CR20] Friel JJ, Terborg R, Langner S, Salge T, Rohde M, Berlin J (2017). X-ray and image analysis in electron microscopy.

[CR21] Fukushima S, Furukawa T, Niioka H, Ichimiya M, Miyake J, Ashida M, Araki T, Hashimoto M (2014). Y2O3:Tm,Yb nanophosphors for correlative upconversion luminescence and cathodoluminescence imaging. Micron.

[CR22] Fukushima S, Furukawa T, Niioka H, Ichimiya M, Sannomiya T, Tanaka N, Onoshima D, Yukawa H, Baba Y, Ashida M, Miyake J, Araki T, Hashimoto M (2016). Correlative near-infrared light and cathodoluminescence microscopy using Y_2_O_3_: Ln, Yb (Ln=Tm, Er) nanophosphors for multiscale, multicolour bioimaging. Sci Rep.

[CR23] Garming MWH, Weppelman IGC, de Boer P, Perona Martínez F, Schirhagl R, Hoogenboom JP, Moerland RJ (2017). Nanoparticle discrimination based on wavelength and lifetime-multiplexed cathodoluminescence microscopy. Nanoscale.

[CR24] Glenn DR, Zhang H, Kasthuri N, Schalek R, Lo PK, Trifonov AS, Park H, Lichtman JW, Walsworth RL (2012). Correlative light and electron microscopy using cathodoluminescence from nanoparticles with distinguishable colours. Sci Rep.

[CR25] Goping G, Pollard HB, Srivastava M, Leapman R (2003). Mapping protein expression in mouse pancreatic islets by immunolabeling and electron energy loss spectrum-imaging. Microsc Res Tech.

[CR26] Hackett MJ, McQuillan JA, El-Assaad F, Aitken JB, Levina A, Cohen DD, Siegele R, Carter EA, Grau GE, Hunt NH, Lay PA (2011). Chemical alterations to murine brain tissue induced by formalin fixation: implications for biospectroscopic imaging and mapping studies of disease pathogenesis. Analyst.

[CR27] Hall TA, Gupta BL (1982). Quantification for the X-ray microanalysis of cryosections. J Microsc.

[CR28] Hell SW (2015). Nanoscopy with focused light (nobel lecture). Angew Chem Int Ed Engl.

[CR29] Hemelaar SR, de Boer P, Chipaux M, Zuidema W, Hamoh T, Martinez FP, Nagl A, Hoogenboom JP, Giepmans BN, Schirhagl R (2017). Nanodiamonds as multi-purpose labels for microscopy. Sci Rep.

[CR30] Herbst R, Hoder D (1978). Cathodoluminescence in biological studies. Scanning.

[CR31] Hoes MF, Beverborg NG, Kijlstra JD, Kuipers J, Swinkels DW, Giepmans BNG, Rodenburg RJ, Veldhuisen, DJv, Boer RAd, Meer Pvd (2018). Iron deficiency impairs contractility of human cardiomyocytes through decreased mitochondrial function. Eur J Heart Fail.

[CR32] Hofer F, Schmidt FP, Grogger W, Kothleitner G (2016). Fundamentals of electron energy-loss spectroscopy. IOP Conf Ser Mater Sci Eng.

[CR33] Hongpaisan J, Roomans GM (1995). Use of post mortem and in vitro tissue specimens for X-ray microanalysis. J Microsc.

[CR34] Jiang H, Kilburn MR, Decelle J, Musat N (2016). NanoSIMS chemical imaging combined with correlative microscopy for biological sample analysis. Curr Opin Biotechnol.

[CR35] Keevend K, Stiefel M, Neuer AL, Matter MT, Neels A, Bertazzo S, Herrmann IK (2017). Tb 3+-doped LaF3 nanocrystals for correlative cathodoluminescence electron microscopy imaging with nanometric resolution in focused ion beam-sectioned biological samples. Nanoscale.

[CR36] Kolinko S, Jogler C, Katzmann E, Wanner G, Peplies J, Schüler D (2012). Single-cell analysis reveals a novel uncultivated magnetotactic bacterium within the candidate division OP3. Environ Microbiol.

[CR37] Leapman RD (2017). Application of EELS and EFTEM to the life sciences enabled by the contributions of Ondrej Krivanek. Ultramicroscopy.

[CR38] Lee RFS, Riedel T, Escrig S, Maclachlan C, Knott GW, Davey CA, Johnsson K, Meibom A, Dyson PJ (2017). Differences in cisplatin distribution in sensitive and resistant ovarian cancer cells: a TEM/NanoSIMS study. Metallomics.

[CR39] Liljesvan B, Roomans GM (1976). Use of pure carbon specimen holders for analytical electron microscopy of thin sections. Ultramicroscopy.

[CR40] Maigne A, Wolf M (2018). Low-dose electron energy-loss spectroscopy using electron counting direct detectors. Microscopy (Oxf).

[CR41] Martin C (2009). GFP: lighting up life (nobel lecture). Angew Chem Int Ed.

[CR42] Mauritz JM, Seear R, Esposito A, Kaminski CF, Skepper JN, Warley A, Lew VL, Tiffert T (2011). X-ray microanalysis investigation of the changes in Na, K, and hemoglobin concentration in plasmodium falciparum-infected red blood cells. Biophys J.

[CR43] Mhawi AA (2009). Interaction of doxorubicin with the subcellular structures of the sensitive and Bcl-xL-overexpressing MCF-7 cell line: confocal and low-energy-loss transmission electron microscopy. Micron.

[CR44] Moriguchi K (2018). Independent trafficking of flavocytochrome b558 and myeloperoxidase to phagosomes during phagocytosis visualised by energy-filtering and energy-dispersive spectroscopy-scanning transmission electron microscopy. J Microsc.

[CR45] Morrison IEG, Samilian A, Coppo P, Ireland TG, Fern GR, Silver J, Withnall R, O’Toole PJ (2015). Multicolour correlative imaging using phosphor probes. J Chem Biol.

[CR46] Nagarajan S, Pioche-Durieu C, Tizei LH, Fang CY, Bertrand JR, Le Cam E, Chang HC, Treussart F, Kociak M (2016). Simultaneous cathodoluminescence and electron microscopy cytometry of cellular vesicles labeled with fluorescent nanodiamonds. Nanoscale.

[CR47] Nagayama K, Onuma T, Ueno R, Tamehiro K, Minoda H (2016). Cathodoluminescence and electron-induced fluorescence enhancement of enhanced green fluorescent protein. J Phys Chem B.

[CR48] Narváez AC, Weppelman IGC, Moerland RJ, Liv N, Zonnevylle AC, Kruit P, Hoogenboom JP (2013). Cathodoluminescence microscopy of nanostructures on glass substrates. Opt Express.

[CR49] Niioka H, Fukushima S, Ichimiya M, Ashida M, Miyake J, Araki T, Hashimoto M (2014). Correlative cathodoluminescence and near-infrared fluorescence imaging for bridging from nanometer to millimeter scale bioimaging. Microscopy (Oxf).

[CR50] Niitsuma J, Oikawa H, Kimura E, Ushiki T, Sekiguchi T (2005). Cathodoluminescence investigation of organic materials. J Electron Microsc.

[CR51] Nisman R, Dellaire G, Ren Y, Li R, Bazett-Jones DP (2004). Application of quantum dots as probes for correlative fluorescence, conventional, and energy-filtered transmission electron microscopy. J Histochem Cytochem.

[CR52] Nuñez J, Renslow R, Cliff JB, Anderton CR (2018). NanoSIMS for biological applications: current practices and analyses. Biointerphases.

[CR53] Paez-Segala MG, Sun MG, Shtengel G, Viswanathan S, Baird MA, Macklin JJ, Patel R, Allen JR, Howe ES, Piszczek G, Hess HF, Davidson MW, Wang Y, Looger LL (2015). Fixation-resistant photoactivatable fluorescent proteins for CLEM. Nat Methods.

[CR54] Pease R, Hayes T (1966). Scanning electron microscopy of biological material. Nature.

[CR55] Pett-Ridge J, Weber PK, Navid A (2012). NanoSIP: NanoSIMS applications for microbial biology. Microbial systems biology: methods and protocols.

[CR56] Philimonenko VV, Philimonenko AA, Sloufova I, Hruby M, Novotny F, Halbhuber Z, Krivjanska M, Nebesarova J, Slouf M, Hozak P (2014). Simultaneous detection of multiple targets for ultrastructural immunocytochemistry. Histochem Cell Biol.

[CR57] Prigozhin MB, Maurer PC, Courtis AM, Liu N, Wisser MD, Siefe C, Tian B, Chan E, Song G, Fischer S, Aloni S, Ogletree DF, Barnard ES, Joubert LM, Rao J, Alivisatos AP, Macfarlane RM, Cohen BE, Ciu Y, Dionne JA, Chu S (2018) Bright sub-20 nm cathodoluminescent nanoprobes for multicolor electron microscopy. arXiv:1806.00075 [cond-mat.mtrl-sci]

[CR58] Ramachandra R, Bouwer JC, Mackey MR, Bushong E, Peltier ST, Xuong NH, Ellisman MH (2014). Improving signal to noise in labeled biological specimens using energy-filtered TEM of sections with a drift correction strategy and a direct detection device. Microsc Microanal.

[CR59] Roomans G, Kuypers G (1980). Background determination in X-ray-microanalysis of biological thin-sections. Ultramicroscopy.

[CR60] Roomans GM, Von Euler A (1996). X-ray microanalysis in cell biology and cell pathology. Cell Biol Int.

[CR61] Sagner S, Kneer R, Wanner G, Cosson J, Deus-Neumann B, Zenk MH (1998). Hyperaccumulation, complexation and distribution of nickel in Sebertia acuminata. Phytochemistry.

[CR62] Saubermann AJ, Beeuwkes R, Peters PD (1981). Application of scanning electron microscopy to X-ray analysis of frozen-hydrated sections. II. Analysis of standard solutions and artificial electrolyte gradients. J Cell Biol.

[CR63] Scotuzzi M, Kuipers J, Wensveen DI, de Boer P, Hagen KC, Hoogenboom JP, Giepmans BN (2017). Multi-color electron microscopy by element-guided identification of cells, organelles and molecules. Sci Rep.

[CR64] Shuman H, Somlyo AV, Safer D, Frey T, Somlyo AP (1983). Applications of energy filtered imaging in biology. Scan Electron Microsc.

[CR65] Somlyo AV, Shuman H, Somlyo AP (1977). Composition of sarcoplasmic reticulum in situ by electron probe X-ray microanalysis. Nature.

[CR66] Somlyo AV, Shuman H, Somlyo AP (1977). Elemental distribution in striated muscle and the effects of hypertonicity. Electron probe analysis of cryo sections. J Cell Biol.

[CR67] Soto M, Zaldibar B, Cancio I, Taylor MG, Turner M, Morgan AJ, Marigómez I (2002). Subcellular distribution of cadmium and its cellular ligands in mussel digestive gland cells as revealed by combined autometallography and X-ray microprobe analysis. Histochem J.

[CR68] Svatoš A (2010). Mass spectrometric imaging of small molecules. Trends Biotechnol.

[CR69] Teng C, Demers H, Brodusch N, Waters K, Gauvin R (2018). Use of an annular silicon drift detector (SDD) versus a conventional sdd makes phase mapping a practical solution for rare earth mineral characterization. Microsc Microanal.

[CR70] Tizei LH, Kociak M (2012). Spectrally and spatially resolved cathodoluminescence of nanodiamonds: local variations of the NV(0) emission properties. Nanotechnology.

[CR71] van der Ent A, Przybyłowicz WJ, de Jonge MD, Harris HH, Ryan CG, Tylko G, Paterson DJ, Barnabas AD, Kopittke PM, Mesjasz-Przybyłowicz J (2018). X-ray elemental mapping techniques for elucidating the ecophysiology of hyperaccumulator plants. New Phytol.

[CR72] Vollnhals F, Audinot J, Wirtz T, Mercier-Bonin M, Fourquaux I, Schroeppel B, Kraushaar U, Lev-Ram V, Ellisman MH, Eswara S (2017). Correlative microscopy combining secondary ion mass spectrometry and electron microscopy: comparison of intensity–hue–saturation and Laplacian pyramid methods for image fusion. Anal Chem.

[CR73] Vrensen GFJM, Marle Jv, Jonges R, Voorhout W, Breipohl W, Wegener AR (2004). Tryptophan deficiency arrests chromatin breakdown in secondary lens fibers of rats. Exp Eye Res.

[CR74] Wilson RG (1995). SIMS quantification in Si, GaAs, and diamond—an update. Int J Mass Spectrom Ion Process.

[CR75] Wirtz T, Philipp P, Audinot JN, Dowsett D, Eswara S (2015). High-resolution high-sensitivity elemental imaging by secondary ion mass spectrometry: from traditional 2D and 3D imaging to correlative microscopy. Nanotechnology.

[CR76] Wu J, Kim A, Bleher R, Myers B, Marvin R, Inada H, Nakamura K, Zhang X, Roth E, Li S (2013). Imaging and elemental mapping of biological specimens with a dual-EDS dedicated scanning transmission electron microscope. Ultramicroscopy.

[CR77] Zierold K, Schäfer D (1978). Quantitative X-ray microanalysis of diffusible ions in the skeletal muscle bulk specimen. J Microsc.

